# What do we know about the effectiveness of the application of hybrid SE–TGfU model in physical education on learning outcomes: a systematic review and meta-analysis

**DOI:** 10.3389/fpsyg.2026.1762732

**Published:** 2026-05-28

**Authors:** Yalin Aygun, Sakir Tufekci, Hulusi Boke, Burak Yagin, Hulya Berktas, Burak Canpolat, Fatih Harun Turhan, Goktug Norman, Emek Guldogan

**Affiliations:** 1 Department of Sport Management, Faculty of Sport Sciences, Inonu University, Malatya, Türkiye; 2 Yasar Oncan Secondary School, Ministry of National Education, Malatya, Türkiye; 3 Department of Biostatistics and Medical Informatics, Faculty of Medicine, Inonu University, Malatya, Türkiye; 4 Department of Physical Education and Sport for Disabled, Faculty of Sport Sciences, Inonu University, Malatya, Türkiye; 5 Department of Sport Management, Hasan Dogan Faculty of Sport Sciences, Karabuk University, Karabuk, Türkiye

**Keywords:** hybrid pedagogical models, models-based practice, physical education, teaching-learning process, traditional skill approach

## Abstract

**Introduction:**

In recent years, there has been growing interest in the hybridization of different pedagogical models (PMs), such as Sport Education (SE) and Teaching Games for Understanding (TGfU), to address underexplored learning outcomes and promote the holistic education of pupils. This study aimed to examine interventions applying a hybrid SE–TGfU model in physical education (PE) to enhance positive learning outcomes in the physical, social, affective, and cognitive domains, and to compare its effects across these domains with those of the Traditional Skill Approach (TSA), isolated SE, and isolated TGfU.

**Methods:**

A systematic search of major scientific databases was conducted. Studies were included if they: (1) implemented the hybrid SE–TGfU model as the instructional intervention, (2) compared it with TSA, isolated SE, or isolated TGfU, and (3) reported quantitative learning outcome(s). After applying the eligibility criteria, a total of nine studies, including 22 effect sizes, were included in the analysis.

**Results:**

The hybrid SE–TGfU model was associated with larger pooled effect sizes than TSA (*g* = 1.29 [0.99; 1.59]) and isolated TGfU (*g* = 0.71 [0.39; 1.04]). No statistically significant difference was observed compared with isolated SE (*g* = 0.97 [−2.05; 3.98]); however, this comparison was based on comparatively limited evidence. Larger pooled effect sizes were observed in three domains, with a distinct ordering from highest to lowest: physical (*g* = 1.75 [0.94; 2.57]), social (*g* = 1.05 [0.57; 1.54]), and affective (*g* = 0.82 [0.67; 0.98]). The effect in the cognitive domain was not statistically significant (*g* = 0.62 [−0.32; 1.57]).

**Discussion:**

The findings suggest that the SE–TGfU hybrid model is associated with larger pooled effect sizes across several learning domains in PE, particularly in the physical, social, and affective domains. However, evidence for the cognitive domain remains inconclusive. These findings should be interpreted cautiously given the substantial heterogeneity and limited evidence base.

**Systematic review registration:**

https://www.crd.york.ac.uk/PROSPERO/view/CRD420251171229.

## 1 Introduction

In the 21-first century, teaching and learning of physical education (PE) at school level continues to occupy a contested space around the world (MacPhail and Lawson, 2020; Calderón and MacPhail, 2023). Scholars and educators have long sought to explore the effectiveness of diverse teaching scenarios in achieving the looked-for educational outcomes in PE classes. Nevertheless, a disconnect between learning processes and teaching strategies persists as a complex and still-unfolding terrain of inquiry (Kirk, 2019). A three-pronged conflict embedded within the traditional skill approach (TSA; Kirk, 2010), commonly referred to as the direct instruction model (Rosenshine, 1976; Engelmann, 1980), have been highlighted: the unattainable pursuit of universal benefits for all learners, the fragmentation of learning through short instructional units, and the inequitable integration of low-skilled students (Casey and Kirk, 2024).

Since the 1970s, the teaching–learning process in PE has progressively shifted from traditional, teacher-centered approaches toward more dynamic and student-centered frameworks (Casey, 2014; López-Lemus et al., 2023). Teaching models (Joyce and Weil, 1972), curriculum models (Jewett and Bain, 1985), and instructional models (Metzler, 2000) were superseded by models-based practice (MbP), an innovative approach to PE that employs distinct pedagogical models (Haerens et al., 2011; Kirk, 2013) in the design and application of the PE program at all grade levels (Casey and Kirk, 2024; Baker, 2025). PMs are described as organizational frameworks that systematically interrelate the interdependent elements of teaching, learning, curriculum and assessment to achieve different learning objectives (Hastie and Casey, 2014). In addition to encouraging reflection on their potential contribution to students’ healthy lifestyle from a physical activity perspective (Casey and Kirk, 2024; Saiz-González et al., 2024b), PMs enhance learning in the physical, social, affective, and cognitive domains (Saiz-González et al., 2024b). Sport Education (SE) and Teaching Games for Understanding (TGfU) are among the most widely acknowledged, implemented, and investigated PMs worldwide (Fernandez-Rio and Iglesias, 2024).

Self-determination theory (SDT; Deci and Ryan, 1985; Ryan and Deci, 2000) also provides a relevant motivational lens for understanding the theoretical foundations of PMs in PE. It describes motivation as a continuum ranging from amotivation to intrinsic motivation, with the latter reflecting volitional engagement driven by interest and enjoyment (Ryan and Deci, 2017). Within PE, intrinsic motivation is associated with adaptive outcomes, including enjoyment, physical activity intentions, and higher physical activity levels, whereas extrinsic motivation is associated with maladaptive outcomes such as boredom and negative affect (Lonsdale et al., 2019; Vasconcellos et al., 2020). Embedded within SDT, basic psychological needs theory posits that individuals have three basic psychological needs: autonomy, competence, and relatedness. These refer, respectively, to the need to self-regulate one’s actions, to feel effective and capable, and to feel socially connected to others (Ryan and Deci, 2017). Environments that support the satisfaction of these needs are more likely to facilitate the internalization of motivation (Vansteenkiste et al., 2020). Accordingly, teaching aligned with SDT may enhance positive learning outcomes in PE, as teacher behaviors that support students’ needs are associated with positive affect and participation, whereas those that undermine competence and relatedness are linked to negative affect and reduced participation (White et al., 2021).

The main idea behind SE is to provide sport experiences that are authentic and educationally meaningful in the context of school PE (Siedentop, 2001) to “educate students to be players in the fullest sense and to help them develop as competent, literate, and enthusiastic sports-people” (Siedentop, 1994, p. 4). To this end, the pedagogy of SE is articulated through six key structural features: seasons, affiliation, formal competition, culminating event, record keeping, and festivity (Siedentop et al., 2019). Enhancing the cultural legitimacy of sport and physical activity while contesting the exclusionary discourses underpinning much of institutionalized sport constitutes a central pedagogical intent within SE (Wallhead and O’sullivan, 2005). Additionally, practice architecture of SE has shown contextual versatility, allowing its principles to be implemented in diverse PE settings (Hastie et al., 2011) as it emphasizes on sport-based content, which remains a central tenet of the PE curriculum worldwide (Kirk, 2013; Casey and Kirk, 2024). While team sports (particularly invasion games) dominate the SE literature, the model is versatile and has been validated for use in racket sports, individual events, and fitness-based activities to achieve its core objectives of developing competent, literate, and enthusiastic sport participants (Siedentop, 1994, 2001; Siedentop et al., 2019).

TGfU was designed by what Harvey et al. (2018, p. 167) refer to as a “games team” (Almond, Bunker, and Thorpe, along with Kirk, Spackman, and others such as Sarah Doolittle, Karen Booth, and Terry Williamson) to shift the focus from technical execution to the tactical understanding of gameplay through modification: representation and exaggeration (Bunker and Thorpe, 1982; Thorpe et al., 1984; Ellis, 1986; Mitchell et al., 2013). The focus is to consider the contextual dynamics of the game (Kirk, 1983) and to develop “intelligent performers in a game” (Almond, 2015, p. 17), “where tactics, decision-making and problem-solving are non-negotiable features, although skill drills are also used to correct any habit or reinforce any skill” (González-Víllora et al., 2021, p. 7). To enhance players’ perception, cognition, and action for a deeper understanding of the game, TGfU is underpinned by six foundational principles: game, game appreciation, tactical awareness, make appropriate decisions, skill execution, and performance (Entwistle, 1969). TGfU is inherently designed for application across all four game categories (e.g., net/wall, striking/fielding, and target games) to achieve its core objectives of developing tactical awareness, decision-making, and game understanding prior to skill execution (Bunker and Thorpe, 1982).

TGfU is built on solid theoretical foundations (i.e., constructivism, guided discovery, and Karl Newell’s ecological theory; Harvey et al., 2018), and gave rise to several derivative frameworks such as Ballschool (Roth and Kröger, 2011), the Developmental Games Stage Model (DGSM; Rink, 2002), Game Sense (GS; den Duyn, 1997; Light, 2014), the Invasion Games Competence Model (IGCM; Mesquita et al., 2012), Play Practice (PP; Launder, 2001), the Tactical-Decision Learning Model (T-DLM; Gréhaigne et al., 2005)or the Tactical Games Approach (TGA; Griffin et al., 1997), coalesced under the umbrella of the Game-Based Approach (Gambles and Gutierrez, 2023).

Although PMs (e.g., SE, TGfU) display their own distinct characteristics, they converge on several common strengths in application (González-Víllora et al., 2019). Joyce and Calhoun (2024) highlight the PMs’ shared common ideas: learning-to-learn competency, learner responsibility and self-regulation, constructivist orientation, scaffolding the learning process, formative assessment and adjustment, collaborative learning and social interaction, holistic skill and competence development, and creativity and reflective thinking. As each PM is designed around particular learning intentions, no single PM can simultaneously address the full range of educational objectives and remain adaptable to the varied realities of PE contexts (Shen and Shao, 2022).

An umbrella review by Fernandez-Rio and Iglesias (2024) points out different weaknesses of PMs that warrant careful consideration by scholars and educators: short units (Araújo et al., 2014; Harvey and Jarrett, 2014), time for skillful play in line with the short units (Araújo et al., 2014; Evangelio et al., 2018), teachers’ limited knowledge/experience (Hastie et al., 2011; Harvey and Jarrett, 2014), problematic or suboptimal performance of student-coaches (e.g., Araújo et al., 2014; Harvey and Jarrett, 2014), and model fidelity (Hastie et al., 2011).

The shared features, inherent limitations, and contextual specificities of PMs (Haerens et al., 2011) have prompted scholars to integrate multiple PMs or selected components thereof to enhance their effectiveness and applicability. Hybridizations between PMs involve the extraction and fusion of key ideas from two PMs, or the use of one as a foundational framework enriched by essential components of the other (González-Víllora et al., 2019). Importantly, Dyson et al. (2004, p. 227) argue that the SE and TGfU models provide students with a situated learning context via “meaningful, purposeful and authentic activities” (Kirk and Macdonald, 1998; Kirk and MacPhail, 2002; MacPhail et al., 2008), which renders them readily compatible for hybrid application.

Although research on hybrid PMs in PE has exponentially increased over the last two decades, reviews addressing the hybridization of PMs remain scarce. As previously outlined, only four reviews have comprehensively examined various hybridizations (González-Víllora et al., 2019; Shen and Shao, 2022; Zhang et al., 2024; Melguizo-Ibáñez et al., 2025). Synthesis of evidence from the first and second systematic reviews underscores the predominance of SE within hybrid PMs, reported in 90 and 88% of the studies, respectively. The third systematic review by Zhang et al. (2024) shows that hybrid PM research is primarily characterized by two dominant models: the combination of SE and TGfU. Herein, SE functions as the structural core, referred to as the “SE + 1 model”, which provides a coherent design architecture for hybridization and has demonstrated synergistic positive effects on students’ learning outcomes. On the other hand, the fourth systematic review with meta-analysis by Melguizo-Ibáñez et al. (2025) provided the first quantitative evidence that both standalone and hybridized PMs enhance basic psychological needs satisfaction and motivational outcomes. Although several hybrid combinations, including SE-TGfU model, were represented among the analyzed interventions, the meta-analysis treated all hybrid PMs as a single analytical construct and found that hybridization generally produced stronger effects on autonomy, competence, and relatedness satisfaction. Further comprehensive and comparative meta-analyses of the effectiveness of the hybridization of SE and TGfU could contribute to a better understanding of the PE literature. To our knowledge, no similar study has been published.

The research questions are:

*RQ1*: To what extent does the hybrid SE–TGfU model improve learning outcomes across the physical, social, affective, and cognitive domains compared with TSA, isolated SE, and isolated TGfU when examined separately, and when considered jointly as a pooled comparison condition?

*RQ2*: How does the effect of the hybrid SE–TGfU model compare with that of TSA?

*RQ3*: How are the effects of the hybrid SE–TGfU model distributed across the four fundamental learning domains?

The research objectives are:

*O1:* To identify experimental and quasi-experimental studies examining the effects of the hybrid SE–TGfU model on PE students’ learning outcomes in the physical, social, affective, and cognitive domains.

*O2:* To compare the effects of the hybrid SE–TGfU model on the physical, social, affective, and cognitive learning domains with those of TSA, isolated SE, and isolated TGfU individually.

*O3:* To examine the effectiveness of the hybrid SE–TGfU model across the physical, social, affective, and cognitive learning domains.

## 2 Methods

### 2.1 Registration and protocol

This systematic review and meta-analysis was conducted and reported in accordance with the PRISMA guidelines (Page et al., 2021). The review protocol was prospectively registered in the PROSPERO database, with the protocol assigned the registration number CRD420251171229.

### 2.2 Eligibility criteria

A PICOS (Population, Intervention, Comparison, Outcomes, and Study Design) framework (McKenzie et al., 2019) was used to structure the reporting of eligibility criteria for reviews of interventions.

#### 2.2.1 Population

Eligible studies were those conducted within PE settings at any educational level and involving student participants. Studies carried out in non-PE contexts or with non-student samples (e.g., teachers, coaches, or adult athletes) were excluded, as their learning processes and pedagogical environments are not comparable to those of students in formal PE settings.

#### 2.2.2 Intervention

Eligible studies were required to implement the hybrid SE–TGfU model as the pedagogical intervention. The intervention had to be systematically executed through a structured instructional framework that operationalized the core tenets of both models (e.g., student-centered learning, tactical understanding, team affiliation, authentic competition). Studies employing a single model (SE or TGfU alone), TSAs, or hybrid PMs other than SE–TGfU were excluded.

#### 2.2.3 Comparison

Eligible studies were required to include at least one comparator group implementing the TSA, SE, or TGfU. Studies lacking a TSA, SE, or TGfU control group, or those employing alternative hybrid pedagogical frameworks, instructional strategies, or methodological designs as comparators were excluded.

#### 2.2.4 Outcomes

Eligible studies were required to report quantitative outcomes reflecting student learning in at least one of the four learning domains (physical, social, affective, and cognitive), consistent with the theoretical framework of this meta-analysis examining the effects of the hybrid SE–TGfU model relative to the TSA, SE, or TGfU. To ensure comparability and enable meta-analysis synthesis, eligible studies had to provide sufficient statistical information to calculate standardized effect sizes (e.g., means, standard deviations, sample sizes, or inferential statistics).

#### 2.2.5 Study design

Eligible studies employed true or quasi-experimental designs incorporating both pre- and post-intervention assessments to assess the effects of the hybrid SE–TGfU model relative to the TSA, SE, and TGfU. Studies were required to report a clearly defined intervention duration, comparison structure, and evaluation framework consistent with established experimental research standards.

### 2.3 Information sources

The literature search was systematically conducted through major electronic databases, registers, and publisher platforms: Web of Science, Scopus, PubMed/MEDLINE, Embase (via Ovid), PsycINFO (via Ovid), Social Policy and Practice (via Ovid), SPORTDiscus, ERIC, EBSCOhost (Education Source, Academic Search Complete, British Education Index, Education Abstracts, Index to Legal Periodicals and Books), ProQuest Central (Education Database, Social Science Database, Applied Social Sciences Index and Abstracts, International Bibliography of the Social Sciences, Sociology Database, Sociological Abstracts), Taylor & Francis Online, SpringerLink, ScienceDirect, and Scielo. Searches were limited to peer-reviewed journal articles and doctoral dissertations published in English, with no publication year restrictions.

In addition, Westlaw UK was consulted to identify policy-related and legal materials relevant to PE practice. Reference lists of all publications eligible for full-text review and relevant systematic reviews were manually screened, and a snowball search was performed using Google Scholar to trace and evaluate studies citing those publications. Supplementary searches were also conducted on websites of relevant professional and research organizations (including governmental departments, educational associations, and research institutes) to capture potentially unpublished or grey literature. The final search for each source was completed on 16 October 2025.

### 2.4 Search strategy

The data search for the present review was conducted using the following descriptors: (*“Sport Education”* OR “Teaching Games for Understanding” OR *“SE–TGfU Hybrid”* OR “Hybrid Pedagogical Model” OR *“Hybrid”* OR *“Integrate”* OR “Combine^*^”) AND “Physical Education^*^.” After completing the database search, all article records were screened, and studies meeting the predefined inclusion criteria were selected for full-text analysis.

### 2.5 Selection process

Two reviewers independently screened all records retrieved from the database searches (Waffenschmidt et al., 2019; Wang et al., 2020). In the first stage, titles and abstracts were screened in duplicate, and disagreements were resolved through discussion. When consensus could not be reached, a third reviewer acted as arbiter. In the second stage, full-text articles were assessed independently by the same two reviewers using the pre-specified inclusion criteria, with unresolved disagreements again resolved by a third reviewer. Cohen’s Kappa was used to study the degree of agreement between two authors (Cohen, 1960). All screening decisions were based solely on human review, and no translations or author queries were required.

### 2.6 Data collection process

A standardized data extraction form was developed prior to coding and applied to all included reports. Two reviewers independently extracted data from each study (Mayo-Wilson et al., 2018), including sample characteristics, group means, standard deviations, sample sizes, intervention features, and outcome measures required to compute effect sizes. Extracted data were compared item-by-item, and discrepancies were resolved through consensus. A third reviewer adjudicated unresolved differences when necessary.

### 2.7 Data items

Data were extracted for learning outcomes in four domains: physical, social, affective, and cognitive. Each domain could include multiple learning outcomes within a study (e.g., motivation, enjoyment, and self-efficacy within the affective domain). When more than one eligible outcome was reported within the same domain, all results were collected and combined into a single domain-level effect size using the Cochrane-recommended data combination formulas (Higgins et al., 2024). Only post-intervention measures were eligible, and no modifications were made to the definitions of the outcome domains during the review.

For each study, data were extracted on sample size, country, participant characteristics, educational stage, number of sessions, learning outcomes, and the intervention–comparator structure. The hybrid SE–TGfU model was the sole intervention of interest, and comparators included TSA, SE, or TGfU conditions. Summary statistics required for effect size computation (group means, standard deviations, and sample sizes) were extracted for both intervention and comparator groups. Study-level effect sizes were examined during synthesis to identify unusually large estimates that could influence pooled results, and their potential impact was considered in the interpretation of overall findings.

### 2.8 Study risk of bias assessment

#### 2.8.1 Assessment of internal validity: Cochrane RoB 2.0

Risk of bias in the included studies was assessed using the revised Cochrane “Risk of bias” tool for randomized trials (RoB 2.0), employing the additional guidance for cluster-randomized and cross-over trials (Eldridge et al., 2016; Higgins et al., 2016). RoB 2.0 assesses five methodological domains: (a) bias arising from the randomization process, (b) bias due to deviations from intended interventions, (c) bias due to missing outcome data, (d) bias in outcome measurement, and (e) bias in the selection of reported results. Following RoB 2.0 guidance (Higgins et al., 2016), two independent reviewers conducted domain-level assessments for each study and documented the corresponding risk-of-bias judgments (“low risk,” “some concerns,” or “high risk”) together with written rationales. Disagreements were resolved through discussion, and when consensus could not be reached, a third reviewer adjudicated. Because the review included both true and quasi-experimental intervention studies, RoB 2.0 was used as a structured framework to assess internal validity across the evidence base. For quasi-experimental studies, judgments were made conservatively, with particular attention to allocation procedures, deviations from intended interventions, outcome measurement, and selective reporting. High-risk judgments in the randomization domain were therefore interpreted as reflecting non-random allocation or insufficient reporting where applicable.

#### 2.8.2 Assessment of methodological rigor: MERSQI

Methodological quality and reporting rigor were assessed using an adapted version of the Medical Education Research Study Quality Instrument (MERSQI) (Reed et al., 2007). The original MERSQI assesses six dimensions: study design, sampling methodology, data collection procedures, validity of assessment instruments, analytical methods, and outcomes evaluation. As the instrument was designed for medical education, clinical outcome items were removed to maintain methodological relevance to PE interventions. This adaptation preserved the conceptual structure of MERSQI while yielding a revised scoring range of 0–17 points (compared to the original 5–18 scale). Each included study received an adapted MERSQI score (study quality score) based on these criteria. The adapted MERSQI was used to evaluate methodological rigor and reporting quality rather than internal validity or domain-specific risk of bias.

### 2.9 Effect measures

As all outcomes were continuous and measured with non-equivalent instruments, standardized mean differences (Hedges’s *g*) were selected as the unified effect metric to ensure commensurable effect estimation. For each study, effect sizes were derived from post-intervention means, standard deviations, and sample sizes (Hedges and Olkin, 2014). Effect magnitude was interpreted in line with guidance from the Cochrane Handbook (Higgins et al., 2024), which considers standardized mean differences of approximately 0.2, 0.5, and 0.8 to be indicative of small, moderate, and large effects, respectively, while recognizing these thresholds as general benchmarks rather than strict cutoffs. In addition to statistical significance, interpretation of pooled effects considered the magnitude of estimates, the width of confidence intervals, and the degree of between-study heterogeneity to support cautious evaluation of result stability.

### 2.10 Synthesis methods

All syntheses were structured around planned pairwise comparisons between the hybrid SE–TGfU model and each comparator condition (TSA, SE, and TGfU) separately. In addition, an overall pooled effect size was calculated in which TSA, SE, and TGfU were considered jointly as a combined comparison condition to provide a complementary summary estimate. Subsequently, subgroup analyses were conducted to examine effect sizes across the four learning domains: physical, social, affective, and cognitive. To determine eligibility for each synthesis, study characteristics were tabulated and cross-checked based on the aforementioned PICOS framework. Only studies implementing hybrid SE–TGfU model as the experimental intervention and providing extractable post-intervention continuous outcomes within at least one of the four learning domains (physical, social, affective, and cognitive) were allocated to a synthesis. Comparator type (TSA, SE, TGfU) served as the grouping variable. Continuous outcome data were standardized prior to synthesis. Summary statistics reported as standard errors were converted to standard deviations when required. All quantitative syntheses were performed by two independent reviewers using Comprehensive Meta-Analysis (CMA) version 4 (Borenstein et al., 2021), which generated standardized mean difference estimates and corresponding 95% confidence intervals. No imputation of missing standard deviations was necessary. Results were visually displayed using forest plots, which depicted individual study effect sizes, confidence intervals, and pooled estimates. Studies were ordered within plots based on effect size magnitude to facilitate visual identification of distributional patterns and potential heterogeneity (Huedo-Medina et al., 2006; Robson et al., 2019).

As conceptual and methodological diversity was expected among included studies, a random-effects model was prespecified and applied in all syntheses (Borenstein et al., 2021). Pooled effects were estimated using the inverse-variance method. Statistical heterogeneity was evaluated through visual inspection of forest plots, Cochran’s *Q*, and the *I^2^* statistic with 95% *CI*s. Heterogeneity was deemed substantial when *I^2^* exceeded 75%, considering *CI* bounds and effect-size dispersion patterns. The DerSimonian–Laird estimator was used to quantify between-study variance (*τ^2^*), and standard Wald-type confidence intervals were applied to pooled effects. Formal sensitivity analyses (e.g., leave-one-out or influence diagnostics) were not performed.

### 2.11 Reporting bias assessment

Risk of bias arising from missing results was appraised through a structured integration of graphical diagnostics and formal statistical tests. Funnel plots were systematically examined for asymmetry indicative of selective publication or the preferential non-reporting of small or null-effect studies. The classic fail-safe *N* (*Nfs*) was computed using Rosenthal’s method to estimate how many unretrieved null-effect studies would be needed to render the pooled effect non-significant. Following Rosenthal’s rule, Nfs values exceeding 5 *k* + 10 (*k* = number of studies) were interpreted as providing limited indication of publication bias, while recognizing that such diagnostics are less informative in small evidence bases (Khoury et al., 2013). Begg and Mazumdar’s (1994) rank-correlation test was used to evaluate the monotonic association between standardized effect sizes and their variances, thereby assessing the presence of selection mechanisms. Egger et al.’s (1997) regression intercept test was employed to detect small-study effects by modeling the linear relation between effect size estimates and their statistical precision.

### 2.12 Certainty assessment

Certainty of evidence was assessed using the GRADE framework (Grading of Recommendations Assessment, Development and Evaluation). The assessment followed GRADE guidance by examining five domains (risk of bias, inconsistency, imprecision, indirectness, and publication bias) and applying predefined decision rules for downgrading (Guyatt et al., 2008). Two reviewers conducted all GRADE judgments independently for each synthesis and outcome category. All domain ratings were documented, and disagreements were resolved through discussion until consensus was reached. This process ensured transparent and reproducible certainty classifications.

## 3 Results

### 3.1 Study selection

**Figure 1:**
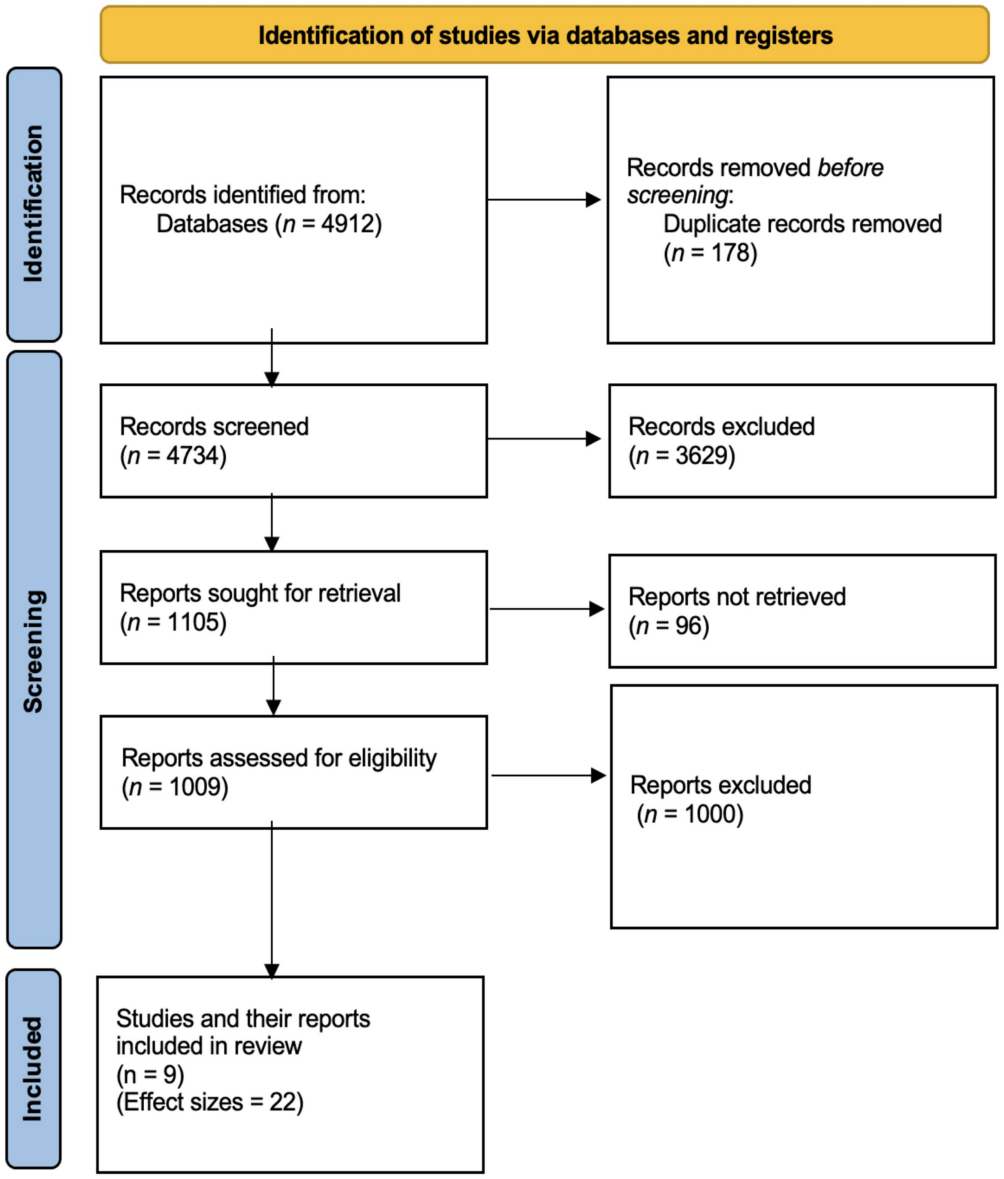
PRISMA flow diagram of the study-selection process (Page et al., 2021).

A total of 4,912 records were identified through the database search. After the removal of 178 duplicate records, 4,734 records remained for screening. Following title and abstract screening, 3,629 records were excluded. The full texts of 1,105 reports were sought for retrieval; however, 96 reports could not be retrieved because they were not available in full text. Consequently, 1,009 full-text reports were assessed for eligibility. After full-text assessment, 1,000 reports were excluded because they did not meet the predefined inclusion criteria and/or did not provide sufficient quantitative data required for meta-analysis. Ultimately, 9 studies were included in the review, contributing 22 effect sizes. In line with the requirements of meta-analysis, only studies reporting the necessary statistical data for both comparison groups, including means, standard deviations, and sample sizes, were eligible for quantitative synthesis (Sánchez-Meca, 2022) The included studies were (Gil-Arias et al., 2017, 2020a, 2020b, 2021; Jani et al., 2017; García-González et al., 2020; López-Lemus et al., 2023; Pan et al., 2023; Ghorbanzadeh et al., 2024; Mahardhika et al., 2024). Interrater agreement between the evaluators was high, with Cohen’s Kappa calculated as 0.83 (Cohen, 1960), indicating strong consistency (Landis and Koch, 1977; Viera and Garrett, 2005). Figure 1 presents the PRISMA flow diagram of the study-selection process.

### 3.2 Study characteristics

The final sample of this meta-analysis consists of 22 individual effect sizes extracted from 9 primary studies, representing a total of 1,363 students in four national contexts (Türkiye, Spain, Indonesia, and Taiwan). As shown in Table 1, most interventions were conducted in secondary education settings (65%; *n* = 13), followed by primary education (20%; *n* = 4), with a smaller proportion implemented in university-level contexts (15%; *n* = 3). Analysis of the learning outcome structure shows that all extracted effects fall into four predefined domains: physical, social, affective, and cognitive. Physical learning outcomes involve motor proficiency and skill-execution measures; social outcomes encompass cooperation, responsibility, and interaction; affective learning outcomes include motivation-, emotion-, and self-efficacy–related indicators; and cognitive learning outcomes capture decision-making and game-performance variables.

**Table 1:** Characteristics of the final study sample.

Authors/dates	Country	Sample	Educational stage	Number of sessions	Study quality score	Comparators relative to SE–TGfU	Learning outcomes	Hedges’s *g*
Ghorbanzadeh et al. (2024)	Türkiye	80 students	Secondary education	16 Sessions	13/17	TSA	Physical motor proficiency	3.587
						SE	Physical motor proficiency	2.523
						TGfU	Physical motor proficiency	1.679
Gil-Arias et al. (2017)	Spain	55 students	Secondary education	16 Sessions	14/17	TSA	Affective autonomy support, satisfaction, autonomous motivation, intention to be physically active	0.730
							Social friendship goals, interaction with others, social dimension within autonomy support	0.846
Gil-Arias et al. (2020a)	Spain	55 students	Secondary education	16 Sessions	14/17	TSA	Affective motivation, emotions, self-efficacy, enjoyment	1.238
							Social team climate, interaction, participation	2.071
Gil-Arias et al. (2020b)	Spain	292 students	Primary education	16 Sessions	15/17	TSA	Affective satisfaction, autonomous motivation, PE satisfaction	0.729
Gil-Arias et al. (2021)	Spain	292 students	Primary education	16 Sessions	15/17	TSA	Affective need for autonomy, need for competence	0.775
							Social need for relationship, belonging	0.857

Authors/dates	Country	Sample	Educational stage	Number of sessions	Study quality score	Comparators relative to SE–TGfU	Learning outcomes	Hedges’s *g*
López-Lemus et al. (2023)	Spain	137 students	Secondary Education	12 Sessions	14/17	TSA	Affective enjoyment, perceived competence, intention to be active	0.925
							Cognitive decision making, game performance, game involvement	2.116
							Physical skill execution	1.649
Mahardhika et al. (2024)	Indonesia	54 students	University	36 Sessions	17/17	TGfU	Affective vigor, dedication, absorption	1.209
							Cognitive decision making, support	1.115
							Physical skill execution	1.254
Pan et al. (2023)	Taiwan	90 students	Secondary education	20 sessions	15/17	TGfU	Affective self-efficacy, interest, value, expectation	0.416
							Cognitive decision making	0.431
							Physical skill practice, support	0.220
							Social responsibility: help, follow rules, cooperation	0.533
Salimin et al. (2020)	Malaysia	96 students	Secondary education	15 sessions	14/17	SE	Cognitive decision making	−0.555
						TGfU	Cognitive decision making	0.000

TSA, Traditional skill approach; SE, Sport education; TGfU, Teaching games for understanding.

### 3.3 Risk of bias in studies

#### 3.3.1 Cochrane RoB 2.0 results

The nine included studies showed a concerning overall risk-of-bias profile, with 4 studies (44%) classified as high risk and 5 studies (56%) rated as having some concerns (Table 2). No study met the criteria for an overall low-risk judgment. The domain most frequently contributing to elevated risk was bias arising from the randomization process, where 5 studies (55%) were judged high risk due to non-random allocation or insufficient reporting. In contrast, missing outcome data presented minimal issues, with 9 studies (100%) rated as low risk. Measurement bias was common, as 7 studies (78%) were rated as having some concerns or high risk, typically due to limited blinding or subjective scoring procedures. Selective reporting issues were also present in all studies (9/9; 100%), each receiving either some concerns or high risk. In sum, while reporting completeness was generally adequate, weaknesses in randomization and outcome measurement produced a moderate-to-high overall risk of bias across the evidence base.

**Table 2:** RoB 2.0 assessment for included studies.

Study	Bias arising from the randomization process	Bias arising from the timing of identification and recruitment of individual participants in relation to timing of randomisation	Bias due to missing outcome data	Bias in measurement of the outcome	Bias in selection of the reported result	Overall risk of bias
Ghorbanzadeh et al. (2024)	Some concerns	Low risk	Low risk	Some concerns	Some concerns	Some concerns
Gil-Arias et al. (2017)	High risk	Some concerns	Low risk	Some concerns	Some concerns	High risk
Gil-Arias et al. (2020a)	Some concerns	Low risk	Low risk	Some concerns	Low risk	Some concerns
Gil-Arias et al. (2020b)	High risk	Some concerns	Low risk	Some concerns	Some concerns	High risk
Gil-Arias et al. (2021)	Some concerns	Low risk	Low risk	Some concerns	Low risk	Some concerns
López-Lemus et al. (2023)	High risk	Some concerns	Low risk	Some concerns	Some concerns	High risk
Mahardhika et al. (2024)	Some concerns	Some concerns	Low risk	Some concerns	Some concerns	Some concerns
Pan et al. (2023)	Low risk	Some concerns	Low risk	Low risk	Some concerns	Some concerns
Salimin et al. (2020)	High risk	Some concerns	Low risk	High risk	Some concerns	High risk

#### 3.3.2 MERSQI results

Based on the adapted MERSQI study quality score (range = 0–17), the included studies demonstrated moderate-to-high methodological quality, with scores ranging from 13 to 17 and an overall mean of 14.6 (Supplementary file, Supplementary Table S1). As the adapted MERSQI assesses methodological rigor rather than domain-specific bias, these scores primarily reflect the methodological soundness of study design, measurement, and analysis. Some limitations were observed in sampling methods and external validity, indicating potential risks related to selection bias and limited generalizability. Overall, the adapted MERSQI scores suggest moderate-to-high methodological rigor in study design, measurement, and analysis; however, these scores should not be interpreted as overriding the internal validity concerns identified by RoB 2.0.

### 3.4 Results of individual studies

Supplementary Figures S1–S3 present the study-level effect sizes (Hedges’s *g*) and corresponding 95% confidence intervals for all comparisons involving the hybrid SE–TGfU model. These include contrasts with TSA, SE, and TGfU examined separately, as well as a combined comparison condition including these instructional models. Effect sizes were derived from post-test group means, standard deviations, and sample sizes, and synthesized using a random-effects model to account for between-study variability. The individual estimates show considerable dispersion, consistent with the heterogeneity values reported in the pooled analyses.

### 3.5 Results of syntheses

#### 3.5.1 Effectiveness of the hybrid SE–TGfU model on learning outcomes compared with a pooled comparison condition including TSA, SE, and TGfU

Supplementary Figure S1 shows that the hybrid SE–TGfU model yields a large pooled effect size on learning outcomes in PE when compared with a pooled comparison condition including TSA, SE, and TGfU (*g* = 1.04; 95% *CI* [0.79, 1.29]; *Q* = 200.72; *I^2^* = 89.54%), although heterogeneity across studies is high.

Subgroup analyses showed domain-specific differences in effect sizes, with the largest effect observed in the physical domain (*g* = 1.75, 95% *CI* [0.94, 2.57]), followed by the social (*g* = 1.05, 95% *CI* [0.57, 1.54]), affective (*g* = 0.82, 95% *CI* [0.67, 0.98]), and cognitive (*g* = 0.62, 95% *CI* [−0.32, 1.57]) domains. The effect estimate for the cognitive domain was not statistically significant.

#### 3.5.2 Effectiveness of the application of hybrid SE–TGfU model on learning outcomes in the physical, social, affective, and cognitive domains compared with the TSA

Supplementary Figure S2 shows that the hybridization of SE and TGfU models yields a large pooled effect size compared with TSA (*g* = 1.29; 95% *CI* [0.99, 1.59]; *Q* = 103.21; I*^2^* = 90.31%; *Z* = 8.43; *p* < 0.001), indicating that the hybrid model is more effective than TSA across the physical, social, affective, and cognitive domains.

#### 3.5.3 Effectiveness of the application of hybrid SE–TGfU model on learning outcomes in the physical, social, affective, and cognitive domains compared with TGfU

Supplementary Figure S3 shows that the hybridization of SE and TGfU models yields a moderate pooled effect size compared with isolated TGfU (*g* = 0.71; 95% *CI* [0.39, 1.04]; *Q* = 32.01; *I^2^* = 75.01%; *Z* = 4.27; *p* < 0.001), indicating that the hybrid model is more effective than isolated TGfU across the physical, social, affective, and cognitive domains.

#### 3.5.4 Effectiveness of the application of hybrid SE–TGfU model on learning outcomes in the physical, social, affective, and cognitive domains compared with isolated SE

Supplementary Figure S4 shows that the hybridization of SE and TGfU models did not yield a statistically significant pooled effect size compared with isolated SE (*g* = 0.97; 95% CI [−2.05, 3.98]; *Q* = 39.64; *I^2^* = 97.48%; *Z* = 0.63; *p* = 0.530). This finding indicates that no statistically reliable difference was observed between the hybrid model and isolated SE. However, this comparison should be interpreted with substantial caution because the evidence base was comparatively weak, comprising only two studies, and heterogeneity was extremely high.

### 3.6 Reporting biases

The funnel plot, fail-safe *N*, Begg and Mazumdar’s test, and Egger’s regression did not provide strong evidence of reporting bias; however, these findings should be interpreted cautiously given the relatively small evidence base. In the funnel plot, the 22 effects were mostly located within the funnel and showed no marked asymmetry around the central axis (Supplementary file, Supplementary Figure S5). The *Nfs* of 2,985 far exceeded the fail-safe criterion of 120 (= [5 × 22] + 10). Begg and Mazumdar’s rank-correlation test indicated a small, non-significant association between effect sizes and their variances (*τ* = 0.28, *p* = 0.067). The intercept of Egger’s regression was not significantly different from zero (intercept = 2.80, *p* = 0.108).

### 3.7 Certainty of evidence

The certainty of evidence was judged to be low (Table 3). This rating was downgraded due to concerns regarding risk of bias, substantial inconsistency, and imprecision. RoB 2.0 indicated that no included study was at overall low risk of bias, with four studies rated as high risk and five as having some concerns. In addition, several pooled analyses showed substantial heterogeneity, consistent with the review’s prespecified threshold for inconsistency. Imprecision was also a concern because the evidence base was small, including only nine primary studies contributing 22 effect sizes, and some domain- and comparator-specific estimates were based on limited data. Although formal publication-bias indicators did not suggest strong asymmetry, these diagnostics were interpreted cautiously given the small number of included studies. Overall, the certainty of evidence was considered low, and the findings should therefore be interpreted with appropriate caution.

**Table 3:** GRADE evidence profile.

GRADE domain	Rating	Justification
Risk of Bias	Moderate	RoB 2.0 indicated important methodological concerns, with 44% of studies rated as high risk and 56% as having some concerns; no study was judged overall low risk. Although MERSQI scores were relatively high (13–17), these reflect methodological rigor at the reporting level and do not override the internal validity concerns identified by RoB 2.0.
Inconsistency	Low	Several pooled analyses showed substantial heterogeneity, with *I^2^* values of 89.54, 90.31, and 75.01%, which is consistent with the review’s prespecified threshold for substantial heterogeneity. Although effect directions were generally similar, the magnitude of effects varied considerably across studies.
Indirectness	Low	The included populations, intervention, comparators, and outcome domains were directly relevant to the review question.
Imprecision	Low–Moderate	The evidence base was small, including only nine primary studies contributing 22 effect sizes. In addition, some comparator- and domain-specific estimates were based on limited data, and some confidence intervals were wide, reducing confidence in the precision and stability of the pooled estimates.
Publication bias	Low	Funnel plot inspection suggested approximate symmetry, Egger’s test was non-significant (*p* = 0.108), Begg’s test was non-significant (*p* = 0.067), and the fail-safe *N* was 2,985, exceeding the conventional threshold of 120. However, these indicators were interpreted cautiously because publication-bias diagnostics are less informative in a small evidence base.

GRADE, Grading of recommendations, assessment, development and evaluation.

## 4 Discussion

The objectives of this study are (a) to examine studies applying a hybrid SE–TGfU model intervention in PE and its effects on positive learning outcomes across the physical, social, affective, and cognitive domains, and (b) to compare the effects of this hybridization across these fundamental domains with those of TSA, isolated SE, and isolated TGfU individually.

Hybridization of SE and TGfU models shows positive effects across several learning domains in PE. Furthermore, the hybrid SE–TGfU model showed larger pooled effect sizes than TSA and isolated TGfU, while no statistically significant difference was observed compared with isolated SE. However, these findings should be interpreted cautiously because the evidence base was small, several pooled analyses showed substantial heterogeneity, and none of the included studies was judged to be at overall low risk of bias. Previous meta-analysis (Melguizo-Ibáñez et al., 2025) and systematic review (González-Víllora et al., 2019; Shen and Shao, 2022; Zhang et al., 2024) studies have found similar results to the present. Melguizo-Ibáñez et al.’s (2025) meta-analysis has shown that hybridization of PMs has a greater effect on learning outcomes (i.e., enjoyment, basic psychological needs). Although most of the studies that form the selected hybridizations are those of SE and TGfU (Zhang et al., 2024; Melguizo-Ibáñez et al., 2025), Melguizo-Ibáñez et al.’s (2025) solitary quantitative synthesis in the literature conflates all hybrid PMs into a single category and does not disentangle the SE–TGfU hybridization from other forms, which restricts the interpretability of its hybrid-model–specific effectiveness.

Regarding the SE–TGfU’s effectiveness, SE, the most extensively used PM (see Fernandez-Rio and Iglesias’s umbrella review in 2024), offers the organization of learners into small, heterogeneous groups (Saiz-González et al., 2024b). This encourages students to provide each other with support in tackling tasks (González-Víllora et al., 2019) and enhance tactical knowledge, skill development, empathy, assertiveness, fair-play, enjoyment, enthusiasm, and motivation (Araújo et al., 2014; Evangelio et al., 2018; Bessa et al., 2019; Sierra-Díaz et al., 2019). The desire to develop competent, literate and enthusiastic people (Siedentop et al., 2019) is directly consonant with SE’s theoretical claims, which are intended to develop sport-specific technique and strategic knowledge, foster responsible leadership, enable effective group functioning, and promote reasoned decision making in sport contexts (Siedentop et al., 2019). SE also has the potential to foster the positive cultural dimensions of sport and physical activity while challenging the exclusionary tendencies of much of institutionalized sport (Wallhead and O’sullivan, 2005). Therefore, SE is a well-established PM endorsed by scholars and educators to achieve the claimed learning outcomes (Fernandez-Rio and Iglesias, 2024).

On the other hand, the use of modified games (representation and exaggeration) (Thorpe et al., 1984; Mitchell et al., 2013), as well as the work on tactical awareness, game appreciation, and making appropriate decisions, with skill execution addressed through corrective and reinforcing practice, are key elements of TGfU (González-Víllora et al., 2021) to improve students’ performance and achieve various positive learning outcomes (e.g., tactical knowledge, skill execution, game performance, physical fitness, personal and social development, attitudes toward physical activity, and motivation) (Miller, 2015; Harvey et al., 2016; Sierra-Díaz et al., 2019; Abad Robles et al., 2020; Barba-Martín et al., 2020; Ortiz et al., 2023). From its inception, TGfU has developed from an educative rather than a sport-science or skill-acquisition orientation. The joint impact of SE and TGfU unites tactical (contextual) understanding with authentic sport experiences (Kirk and MacPhail, 2002). Moreover, the theoretical development of both PMs drew on situated learning and situated cognition and enactivism (Varela et al., 1992; Hopper, 1998; Light, 2017), nonlinear pedagogy and dynamic systems theory (Pill, 2007; Breed and Spittle, 2011; Chow et al., 2013), and the ecological theory of Karl Newell by (Piggott, 1982). These shared and complementary characteristics allow their integration to function effectively in school-based PE. TSA reduces learners’ basic psychological needs (Sierra-Díaz et al., 2019; Viciana et al., 2020) and suppress decision-making and problem-solving, giving rise to weak skill transfer and suboptimal performance outcomes (Harvey and Jarrett, 2014; Bessa et al., 2019). This direct instruction model (Rosenshine, 1976; Engelmann, 1980) positions learners as passive participants in the teaching–learning process, leading to diminished educationally beneficial outcomes (Bessa et al., 2021; Manninen et al., 2025).

Specifically, the present meta-analysis indicates that the largest pooled effect size of the hybrid SE–TGfU model was observed in the physical domain, followed by the social and affective domains, whereas the cognitive domain did not show statistically reliable effects (the estimate remained statistically non-significant across comparisons). The hybridization of SE and TGfU can cause improvements in students’ technical-tactical performance and knowledge of various sports as they decide on strategies together (Stran et al., 2012; Gil-Arias et al., 2017). These shared characteristics allow intragroup and intergroup relationships to develop (Menéndez Santurio and Fernández-Río, 2016; Gil-Arias et al., 2020a). A greater effect on intention to be physically active, autonomous and intrinsic motivation by encouraging students to make decisions that affect the final outcome after the application of this hybrid model is noted (Gil-Arias et al., 2017, 2021; López-Lemus et al., 2023). Open-ended tasks strongly support the development of students’ autonomy (Saiz-González et al., 2024a) and reduce levels of amotivation (Melguizo-Ibáñez et al., 2025). This is mainly due to the promotion of competence when performing any task (García-González et al., 2020). An increased competence is a strong determinant of intrinsic motivation (Gil-Arias et al., 2020a, 2021). When a student acquires a higher degree of competence when performing a task, the task may feel more rewarding because of the enjoyment it generates (Gil-Arias et al., 2017, 2020a; López-Lemus et al., 2023; Pan et al., 2023).

Previous studies report improvements in divergent thinking, decision-making processes, game performance, and physical literacy following SE–TGfU implementation; however, the cognitive-domain estimate in the present meta-analysis was not statistically significant (McCaughtry et al., 2004; López-Lemus et al., 2023; Mahardhika et al., 2024). Indeed, students internalize the governing principles, rules, and structural logics of games and apply them flexibly, most clearly when they transferred these concepts to the novel games they designed by incorporating risk–reward reasoning and strategic skill selection (Hastie and Curtner-Smith, 2006). To successfully achieve one of these outcomes, participants must build their own knowledge with the help of others, scaffold the learning process through on-going formative assessment, and propose different solutions, encouraging critical thinking, creativity and autonomy (González-Víllora et al., 2019; Shen and Shao, 2022; Fernandez-Rio and Iglesias, 2024; Zhang et al., 2024; Melguizo-Ibáñez et al., 2025). Since students’ cognitive learning relies on peers, and some may struggle to fulfil the role effectively, teachers must assist students through the use of aids (e.g., learning cues) and individualized guidance (e.g., feedback) (González-Víllora et al., 2019; Fernandez-Rio and Iglesias, 2024). In this type of hybridization, tactical (contextual) understanding requires coordinated motor execution under authentic game constraints (Hastie and Curtner-Smith, 2006). In the same vein, most studies examining the hybridization of SE and TGfU have reported greater improvements in physical activity levels, as students are made to feel more important in their own motor learning process (Quintas et al., 2020), as well as more refined skill execution and stronger game-related behaviors. These outcomes appear to be promoted by student-centered teaching approaches and the delegation of responsibilities by the teacher within authentic, participatory sporting environments (Hastie and Curtner-Smith, 2006; Stran et al., 2012; Guijarro et al., 2021; Jia, 2021). Nevertheless, girls tend to show lower levels of high-intensity physical activity (MVPA), showing that the hybrid model may not equalize these levels (Oliveros and Fernandez-Rio, 2022).

In recent years, even though there has been a growing interest in the hybridization of SE and TGfU models, there are several gaps because of the novelty of the framework (González-Víllora et al., 2019; Shen and Shao, 2022; Zhang et al., 2024). Some scholars caution that hybridization should not be adopted as a pedagogical trend without sufficient contextual justification (Fernandez-Rio and Iglesias, 2024). Teachers should seek support when attempting to hybridize SE and TGfU.

### 4.1 Limitations and applicability of research

This systematic review and meta-analysis should be interpreted considering several important limitations. First, the overall evidence base was small, comprising only nine primary studies and 22 effect sizes. This constitutes a limited basis for a meta-analysis examining multiple pedagogical contrasts and multiple learning domains. As a result, the statistical power of the analyses was constrained, comparator-specific and domain-specific estimates were less stable, and publication-bias diagnostics were less informative than they would be in a larger evidence base. In addition, pooled estimates may have been disproportionately influenced by a small number of studies reporting relatively large effects.

The limited number of studies was particularly important for some comparator conditions, especially the isolated SE comparison, where the evidence base was especially thin. Accordingly, conclusions drawn for these comparisons should be regarded as preliminary rather than definitive. Similarly, the distribution of primary studies across learning domains was uneven, with relatively limited evidence for the cognitive domain, which restricted the interpretability of domain-specific findings and reduced confidence in that part of the synthesis.

An additional important limitation concerns the overall risk-of-bias profile of the included studies. None of the included studies was judged to be at overall low risk of bias; instead, all studies were rated as either high risk or as having some concerns. This means that the evidence base is limited not only by its small size and heterogeneity, but also by nontrivial methodological concerns across all included studies. As a result, the pooled findings should be interpreted as suggestive rather than definitive.

A limitation should also be acknowledged regarding intervention dosage. Although the number of sessions was extracted and is reported in Supplementary Table S1, the duration of individual teaching lessons was not consistently reported across the included studies, which limits conclusions regarding the practical implementation of the hybrid model in school PE settings.

Further, heterogeneity in study settings, intervention implementation, comparison conditions, and outcome measurement reduced the precision and generalizability of the pooled estimates. Although no time restrictions were applied during the search process, the review was limited to English-language peer-reviewed studies and doctoral dissertations, which may have excluded relevant grey literature or non-indexed studies. Taken together, these limitations suggest that the present findings should be interpreted cautiously and that further high-quality primary studies are needed before firm conclusions can be drawn regarding the comparative effectiveness of the hybrid SE–TGfU model across domains and comparator conditions.

Based on the effect sizes across learning domains, the hybrid SE–TGfU model appears most applicable to PE contents that combine physical engagement with social interaction and affective involvement. In this respect, game-centered and team-based units, particularly invasion games, seem to offer the most compatible context for implementation, as they allow the integration of skill execution, teamwork, affiliation, and motivational engagement. Although the cognitive domain also favored the hybrid model, this effect was not statistically significant and should therefore be interpreted cautiously.

## 5 Conclusion

The current evidence suggests potentially favorable effects of the hybrid SE–TGfU model, particularly relative to TSA; however, the magnitude, precision, and generalizability of these effects are limited by substantial between-study heterogeneity. The pooled analyses suggest larger effect sizes for the hybrid SE–TGfU model than for standalone TSA and isolated TGfU, whereas no statistically reliable difference was found compared with isolated SE. The largest pooled contrast was observed in comparisons with TSA. Pooled effect sizes were largest in the physical domain, followed by the social and affective domains, whereas evidence for the cognitive domain remained inconclusive.

## Data Availability

The original contributions presented in the study are included in the article/Supplementary material, further inquiries can be directed to the corresponding author.
