# Supplementary material for: What do we know about the effectiveness of the application of hybrid SE–TGfU model in physical education on learning outcomes: a systematic review and meta-analysis
Source: Front Psychol. 2026 May 28;17:1762732. doi: 10.3389/fpsyg.2026.1762732 (PMC13253795; doi:10.3389/fpsyg.2026.1762732)
Supplement: Supplementary file 1 [file Supplementary_File_1.docx]

Supplementary Material

# Supplementary Figures

## Supplementary Figures


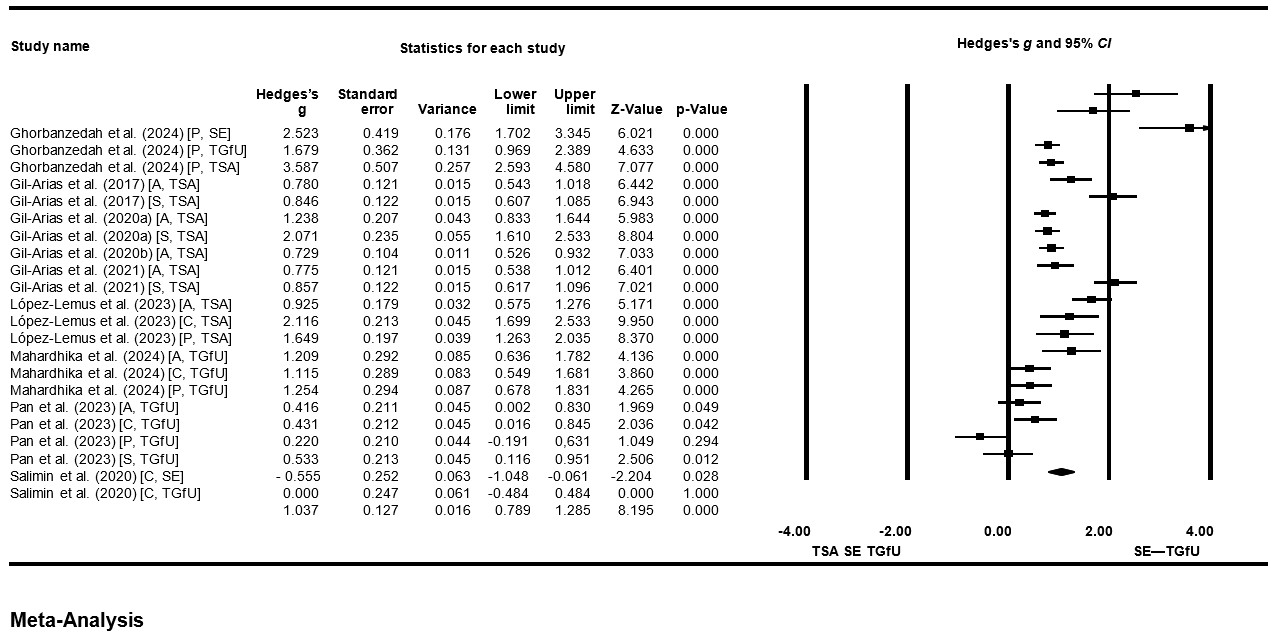


**Supplementary Figure 1.** Effectiveness of the application of hybrid SE–TGfU model on learning outcomes in the physical, social, affective, and cognitive domains compared with a pooled control set in which TSA, SE, and TGfU were each applied separately as single-method control conditions.

*Note.* P = Physical domain; C = Cognitive domain; A = Affective domain; S = Social domain; TSA = Traditional Skill Approach; SE = Sport Education; TGfU = Teaching Games for Understanding.


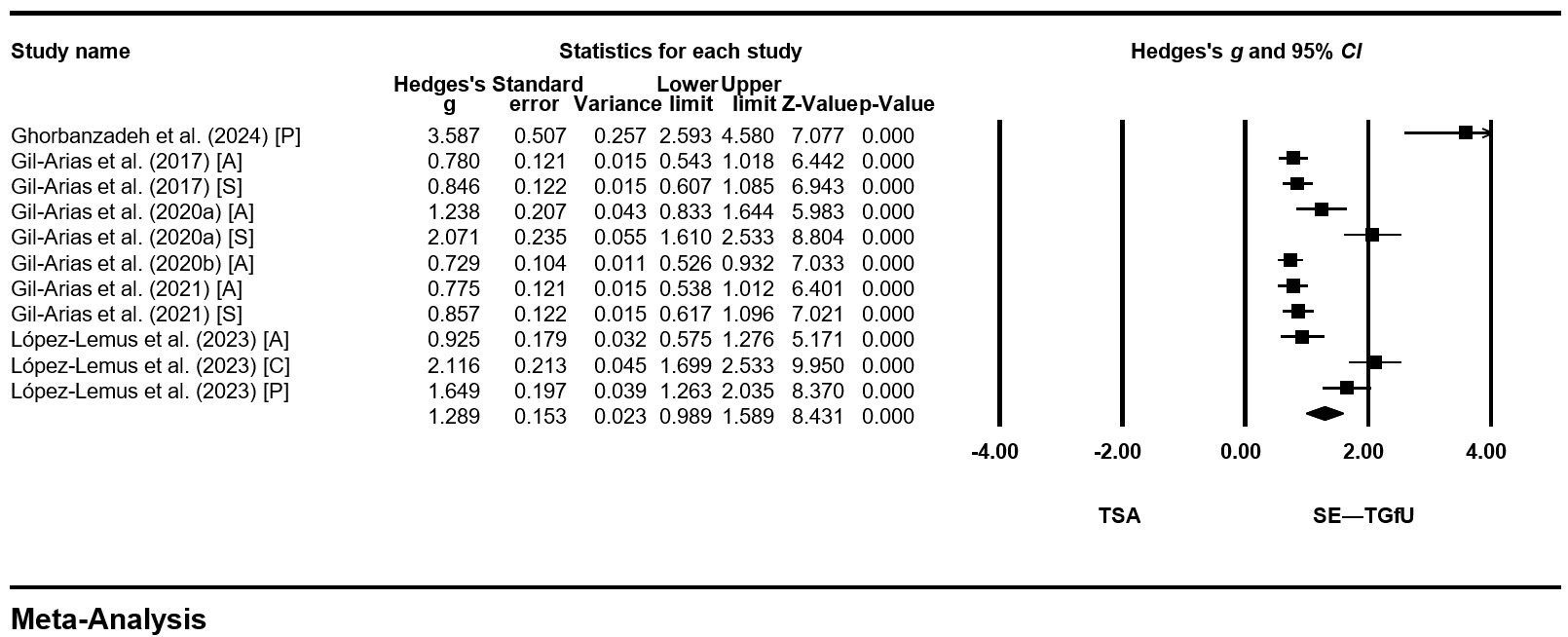


**Supplementary Figure 2.** Effectiveness of the application of hybrid SE–TGfU model on learning outcomes in the physical, social, affective, and cognitive domains compared with the TSA.

*Note.* P = Physical domain; C = Cognitive domain; A = Affective domain; S = Social domain; TSA = Traditional Skill Approach; SE = Sport Education; TGfU = Teaching Games for Understanding.


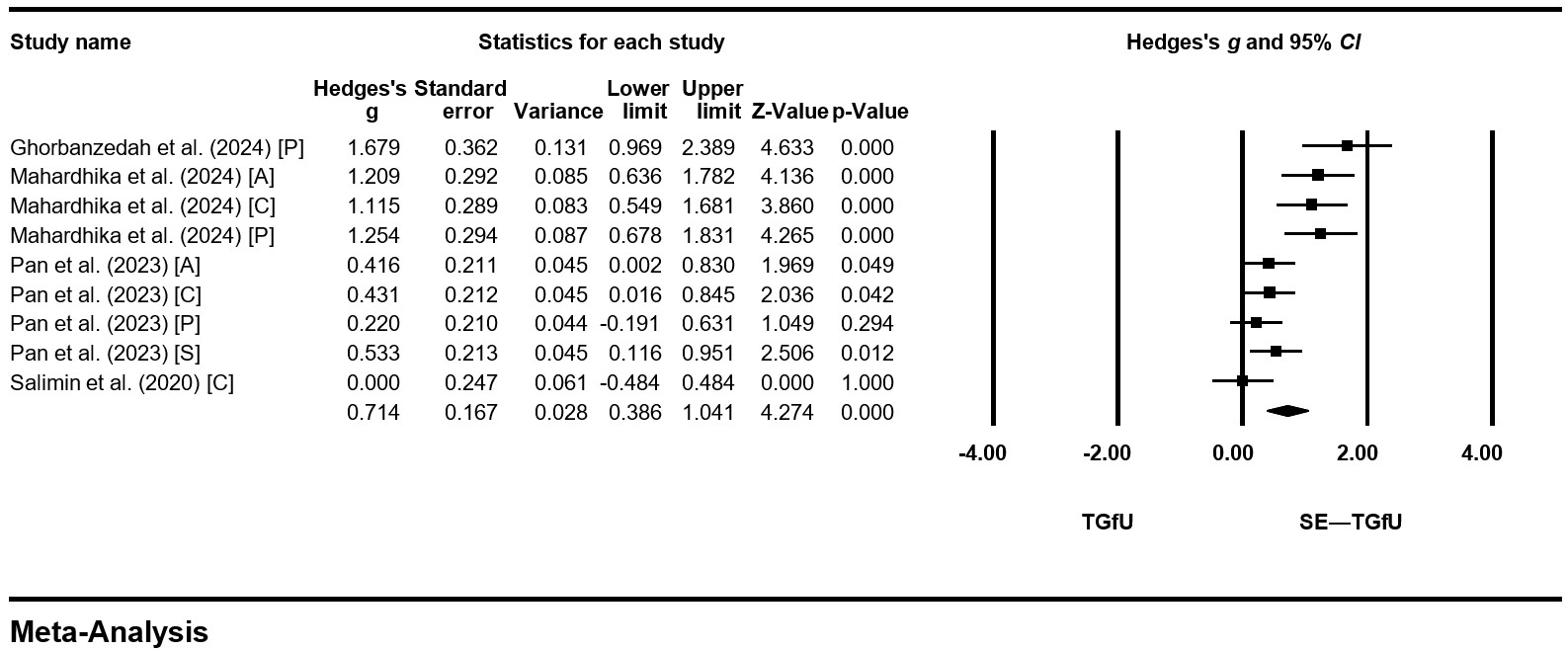


**Supplementary Figure 3.** Effectiveness of the application of hybrid SE–TGfU model on learning outcomes in the physical, social, affective, and cognitive domains compared with TGfU.

*Note.* P = Physical domain; C = Cognitive domain; A = Affective domain; S = Social domain; SE = Sport Education; TGfU = Teaching Games for Understanding.


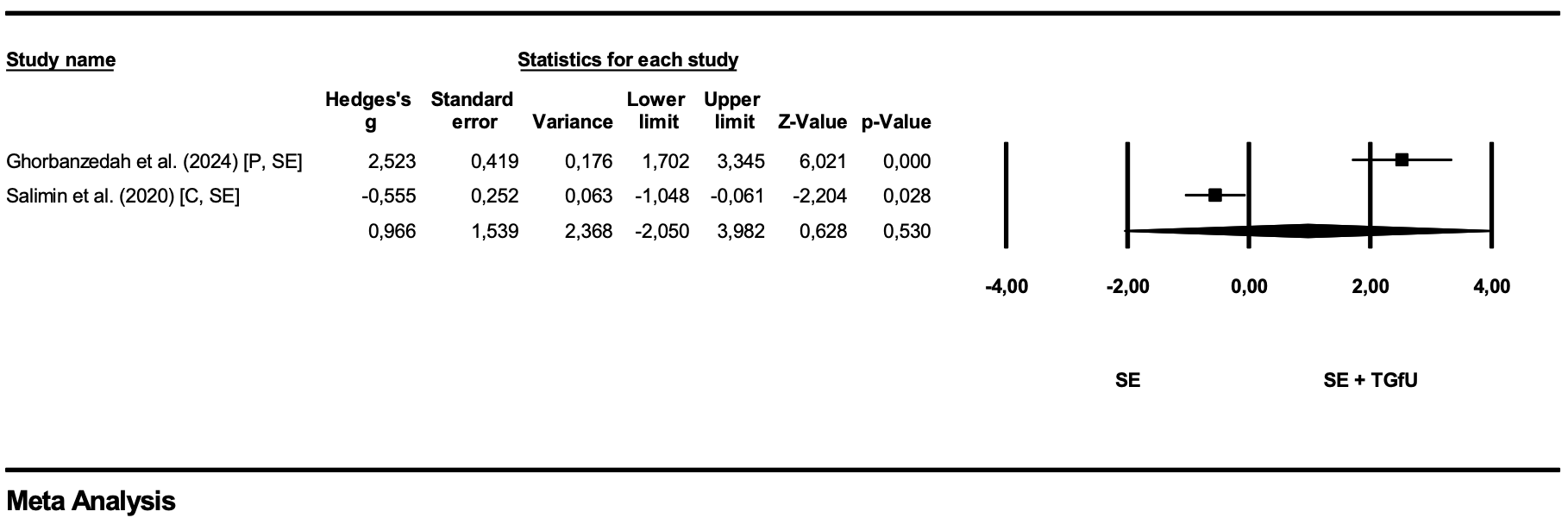


**Supplementary Figure 4.** Effectiveness of the application of hybrid SE–TGfU model on learning outcomes in the physical, social, affective, and cognitive domains compared with isolated SE.

*Note.* P = Physical domain; C = Cognitive domain; A = Affective domain; S = Social domain; SE = Sport Education; TGfU = Teaching Games for Understanding.


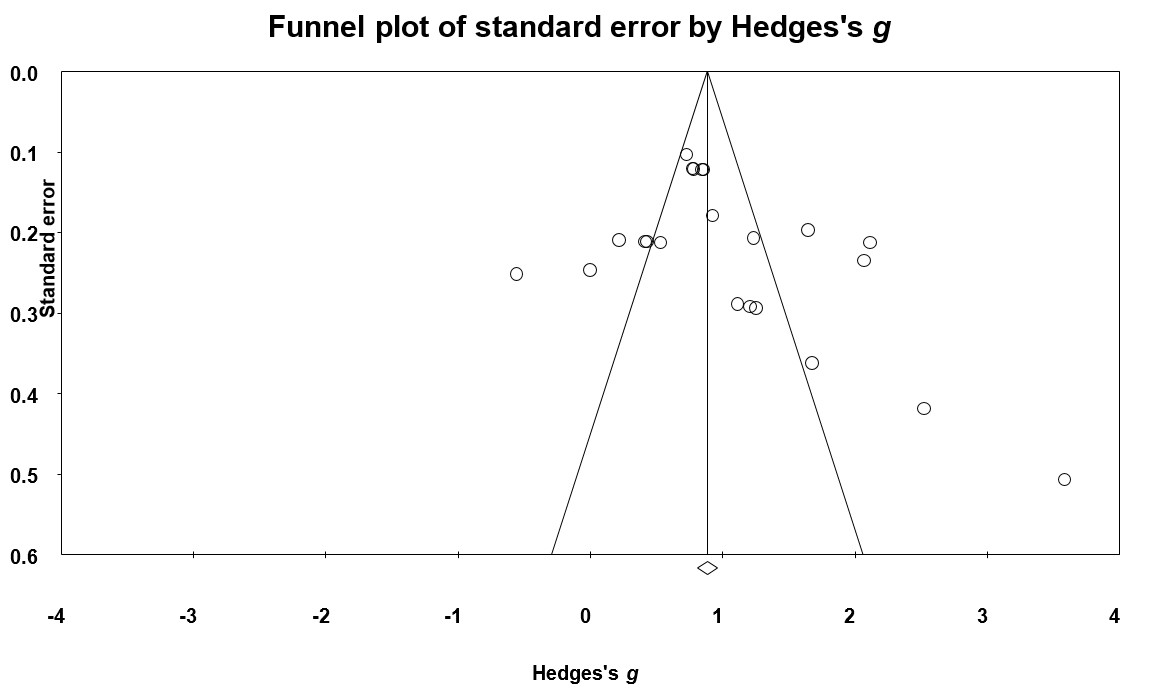


**Supplementary Figure 5.** Funnel plot of effect sizes for learning outcomes in the physical, social, affective, and cognitive domains.
